# Development of the Assessment of Belief Conflict in Relationship-14 (ABCR-14)

**DOI:** 10.1371/journal.pone.0129349

**Published:** 2015-08-06

**Authors:** Makoto Kyougoku, Mutsumi Teraoka, Noriko Masuda, Mariko Ooura, Yasushi Abe

**Affiliations:** 1 Department of Occupational Therapy, School of Health Sciences, Kibi International University, Okayama, Japan; 2 Doctor Course, Graduate School of Health Sciences, Kibi International University, Okayama, Japan; 3 Oosugi Hospital, Okayama, Japan; 4 Kyouseikai Sakurai Hospital, Tokyo, Japan; 5 Graduate School of Health Sciences, Okayama University, Okayama, Japan; 6 Department of Palliative Care, Asahikawa Medical University Hospital, Hokkaido, Japan; Duke University Medical Center, UNITED STATES

## Abstract

**Purpose:**

Nurses and other healthcare workers frequently experience belief conflict, one of the most important, new stress-related problems in both academic and clinical fields.

**Methods:**

In this study, using a sample of 1,683 nursing practitioners, we developed The Assessment of Belief Conflict in Relationship-14 (ABCR-14), a new scale that assesses belief conflict in the healthcare field. Standard psychometric procedures were used to develop and test the scale, including a qualitative framework concept and item-pool development, item reduction, and scale development. We analyzed the psychometric properties of ABCR-14 according to entropy, polyserial correlation coefficient, exploratory factor analysis, confirmatory factor analysis, average variance extracted, Cronbach’s alpha, Pearson product-moment correlation coefficient, and multidimensional item response theory (MIRT).

**Results:**

The results of the analysis supported a three-factor model consisting of 14 items. The validity and reliability of ABCR-14 was suggested by evidence from high construct validity, structural validity, hypothesis testing, internal consistency reliability, and concurrent validity. The result of the MIRT offered strong support for good item response of item slope parameters and difficulty parameters. However, the ABCR-14 Likert scale might need to be explored from the MIRT point of view. Yet, as mentioned above, there is sufficient evidence to support that ABCR-14 has high validity and reliability.

**Conclusion:**

The ABCR-14 demonstrates good psychometric properties for nursing belief conflict. Further studies are recommended to confirm its application in clinical practice.

## Introduction

Job stress is recognized worldwide as a major problem for both worker and organizational health by the International Labor Office [1]. The World Health Organization has defined job stress as “the response people have when presented with work demands and pressures that are not matched to their knowledge and abilities and which challenge their ability to cope” [2]. There has been interest in job stress, especially among healthcare workers, because it is one of the most common work-related health problems in healthcare [3].

One cause of job stress for healthcare workers is conflict [4], which occurs between healthcare workers in the same position (peers), between healthcare workers and other staff members, and between the healthcare team members and the patient or patient’s family [5]. Additionally, high stress is due to healthcare workers having to respond very quickly to complex and various needs of patients and families [6]. In particular, nurses experience considerable stress [7], since they have a wide range of duties, long working hours, and complicated relationships with physicians, other healthcare workers, as well as patients and their families [8].

In Japan, “belief conflict” is one of the hottest new stress-related problems for nurses and other healthcare workers in both academic and clinical fields [9, 10]. Nurses, especially, tend to fall into belief conflicts, as observed by many previous studies [9–12]. Beliefs are units of thinking and doing that nurses do not question [13]. *Belief conflict* is defined as a fundamental confrontation caused by situations individuals face when their beliefs are questioned [13]. For example, belief conflict might arise because of differences in opinion on interventions or because of a medical worker and a patient’s family misunderstanding the patient’s desire to be heard. Such belief conflicts with healthcare workers, patients, and their families have been significant to job stress [14].

Over the past few years, several studies have been conducted on belief conflict in healthcare, but one important study, the Dissolution Approach for Belief conflict (DAB), was developed through theoretical and practical studies in 2011 [13, 14]. DAB is a comprehensive intervention program aiming, as its ultimate goal, to support those suffering from belief conflicts [14]. This model is characterized by “dissolution,” which refers to clarification of belief conflict [15]. Namely, dissolution signifies resolution and disappearance of the problem [16]. Compared with other medical conflict models, the dissolution principle is a prominent characteristic unique to DAB, inspired by Structure Constructivism [17], Phenomenology [18], Constructivism [19], and a Zen Buddhism-based Dissolution-Oriented Approach [13].

In Japan, this model has been developed, applied, and refined by the developer himself and by other healthcare workers [13, 14]. For example, DAB has been applied in situations such as care of an unstable person, support of healthcare workers suffering from conflict, improvement of inter-professional work/education, promotion of therapeutic relationships, and conflict between eastern and western medicine [13–15, 20]. Moreover, DAB is applied in diverse circumstances, for example, in hospitals, outpatient clinics, nursing homes, rehabilitation programs, and organizations [13–15]. Thus, DAB was developed for people experiencing belief conflicts in their practices and was designed to apply to a variety of contexts.

Moreover, DAB-related experimental studies indicated that nurses and other healthcare workers were experiencing strong belief conflict [21–24], a main factor fraught with the difficulties of multidisciplinary care. Belief conflict was associated with an increase in the risk of job stress and other negative emotions (e.g., anger, grief, anxiety, dilemma) [25, 26]. Clearly, nurses and other healthcare practitioners could slip into a vicious cycle of belief conflict, which is specifically conceptualized as a job-related condition and generally, as a human-relationship problem. Furthermore, case study results indicated that DAB was clinically useful for overcoming belief conflict [27], and that DAB worked better in combination with mind mapping [28]. At present, however, there are no comprehensive, validated assessments for measuring nurses’ ideas regarding belief conflict.

Therefore, we organized a research group to develop a new, belief-conflict-specific, psychometrically valid, self-reported measure, named the Assessment of Belief Conflict in Relationship (ABCR), on the basis of DAB. This model’s perspective can be identified with a comprehensive concept framework of belief conflict in human relationships at work, and from this, it can create problem-related item pools. This development is specifically significant for healthcare professionals, because it enables quantitative evaluation of belief conflict.

## Methods

The ABCR development methodology included informed consent, development of a construct framework and an item pool, item reduction, and development of assessment.

### Ethics statement

The Ethics Committee of Kibi International University approved this study (No. 13-01). In this study, along with a survey form, we enclosed a letter explaining the purpose and method, and informed consent. All participation was voluntary, and participants had the right to leave the study at any time without providing a reason. We regarded the return of the survey form as consent for participation. The survey form was returned anonymously in sealed submission envelopes.

### Development of framework concept and item pools

The ABCR item pool was generated through a multi-step process: (1) constructing a framework concept, (2) reviewing relevant assessments, and (3) convening an expert panel.

DAB was used as a construct framework concept in ABCR development, because it has a theoretical and practical foundation for resolving belief conflict. The framework concept for ABCR was defined on the basis of a DAB literature review by literature researcher [13–15]. For over a decade in total, this literature researcher had a background as an educator and researcher of DAB in Graduate School of Nursing Master and Doctor Courses in two universities, and had a clinical experience in hospital and facility. That is, the reviewer of literature was expert in nursing related belief conflict and DAB. As a result, three belief conflicts that arise in human relationships were considered: (1) among the same healthcare professionals, defined as conflict arising from a perception gap among peers; (2) between healthcare workers and other staff, defined as conflict arising from a perception gap among different healthcare practitioners; (3) in therapeutic relationships, defined as conflict arising from a perception gap between the patient/family and the healthcare professional.

Moreover, the above literature researcher and three experts gathered an initial pool of 45 items by using this framework: (1) belief conflict among the same healthcare workers (15 items); (2) belief conflict between healthcare workers and other staff (15 items); and (3) belief conflict in therapeutic relationships (15 items). The newly involved three experts had clinical experience of nurse home, rehabilitation, and DAB in hospital (average years of clinical experience (standard deviation, SD) = 10.667 (5.859)). The desired number of items for the final ABCR scale was 10 to 15. Relevant assessment included the Emotion Labor Inventory [29], Stressor/Stress Reaction Scales [30], Scale of Nurses’ Job Satisfaction [31], Nursing Stressor Inventory [32], and many other assessments. The ABCR item design was based on a 7-point Likert scale in which 1 corresponded to *strongly disagree*; 2 to *disagree*; 3 to *slightly disagree*; 4 to *neither agree nor disagree*; 5 to *slightly agree*; 6 to *agree*; and 7 to *strongly agree*.

To assess the instrument’s face and content validity, a panel of seven DAB experts reviewed the item pool. The expert panel had the licentiate of five occupational therapists, and two nurses. All paneler had clinical experience in hospital such as emergency medicine, orthopedic, psychiatry, palliative care (average years of clinical experience (SD) = 12.857 (7.559)). The ABCR was developed using the expert consensus method in three steps: Step 1: item pool screening; Step 2: item adjustment; Step 3: final review. In Step 1, the item pool was screened to determine the question type and the content validity of the framework concepts. Using a Likert scale of 1–2 (1 = least plausible; 2 = most plausible), 45 items were rated for importance. For inclusion in the ABCR item pool, 85% consensus of the expert panel was needed. If the Likert rating was 1, the panelist was asked to suggest additional exemplars, modifications of wording, or deletions, as appropriate. Step 2 included language modifications of the collected questions to reflect the results of the previous step. In Step 3, the expert panel again reviewed the 45 items to ensure that the belief-conflict-related framework concept was adequately represented in the draft. After Step 3, nurses and literature researcher checked final details on framework concept and item pools. The 45 items successfully screened through this process were sent for field-testing. The final ABCR pool contained 45 belief-conflict items.

### Item reduction and development of scales

#### Participants

Participants were recruited through cooperation with research collaborators in Japan. A non-random sample of 2,951 nurses and allied health professionals were contacted in 11 hospitals.

#### Additional Measures

In addition to the 45-item ABCR version, participating nurses were also assessed using the following measures.

#### Job Content Questionnaire

Job stress was measured using the Job Content Questionnaire (JCQ), developed by Karasek et al., based on the demand-control-support model [33, 34]. JCQ contains 22 items on three scales: job demands (5 items; score range, 12–48), job control (9 items; score range, 24–96), and worksite social support (8 items; score range, 8–32), with a four-point response from 1 (strongly disagree) to 4 (strongly agree).

#### Participant Profile

Demographic data were obtained from participants. We assessed gender, age, years of clinical experience, licensing, position, working arrangements, vacation, leaves of absence, health condition, team approach to healthcare, marriage, smoking, and drinking alcohol.

### Statistical Analysis

We conducted analyses using SPSS Statistics Version 22 (http://www.spss.com), Exametrika 5.3 (http://antlers.rd.dnc.ac.jp/~shojima/exmk/index.htm), Mplus Version 7.2 (http://www.statmodel.com).

### Sample Characteristics

The participants’ demographics and JCQ were summarized using descriptive analyses in SPSS Statistics 22.

#### Item reduction

Of the 45 items, some were deleted according to certain conditions of item analysis: (1) item floor effect or ceiling effect was confirmed in SPSS Statistics 22; (2) entropy of item was much smaller in Exametrika 5.3 (entropy < 1.5); (3) polyserial correlation coefficient between each item and the total score, excluding the item, was very low compared with that of other items in Exametrika 5.3 (polyserial correlation coefficient < 0.2).

#### Construct validity

The ABCR’s item reduction and factor structure was determined by performing exploratory factor analysis (EFA), using a robust weighted least squares factoring method with missing data (WLSMV) in Mplus 7.2 [35]. Items were also considered for deletion on the basis of framework concepts: (1) belief conflict among the same healthcare workers; (2) belief conflict between healthcare workers and other staff; (3) belief conflict in therapeutic relationships. Items not loading on a factor (factor loading < 0.4) or loading on more than one factor were deleted from the scale; analysis was then conducted with the reduced item set. Percentage of variance in the items accounted for by a factor was estimated using eigenvalues. We used three indexes to assess model data fit [36, 37]. The first index was the root mean square error of approximation (RMSEA). The critical values of RMSEA from 0.08 to 0.10 show a mediocre fit, and below 0.08 indicates a good fit [38]. The second and third indexes were the comparative fit index (CFI) and the Tucker–Lewis index (TLI), both with critical values above 0.95 [39].

#### Structural validity

We performed an EFA followed by a confirmatory factor analysis (CFA). The CFA was performed with WLSMV in Mplus 7.2. Model data fit assessment of the CFA was based on multiple indicators, including RMSEA, CFI, and TLI. Values smaller than 0.08 for RMSEA are considered an acceptable and ideal fit [38]. Values greater than 0.90 for CFI and TLI are considered adequate although values approaching 0.95 are preferable [39].

#### Hypothesis testing

We analyzed to address the discriminant and convergent validity, based on the factor structure supported by CFA of ABCR-14. To substantiate the evidence of convergent and discriminant validity, we calculated the square of correlation between factors, average variance extracted (AVE) for ABCR in SPSS Statistics 22 and Mplus 7.2. AVE measures the explained variance of the construct [40]. To check convergent validity, we tested whether the square root of every AVE value belonging to each latent construct was greater than 0.5. Discriminant validity was assessed by comparing the squared correlation between each pair of constructs against the average of the AVE for the ABCR factor structure.

#### Internal consistency reliability

Internal consistency reliability was assessed using Cronbach’s alpha in SPSS Statistics 22.

#### Concurrent validity

We analyzed the Pearson product-moment correlation coefficient of the between factor score of both ABCR and JCQ in SPSS Statistics Version 22.

#### Item response

**Fig 1:**
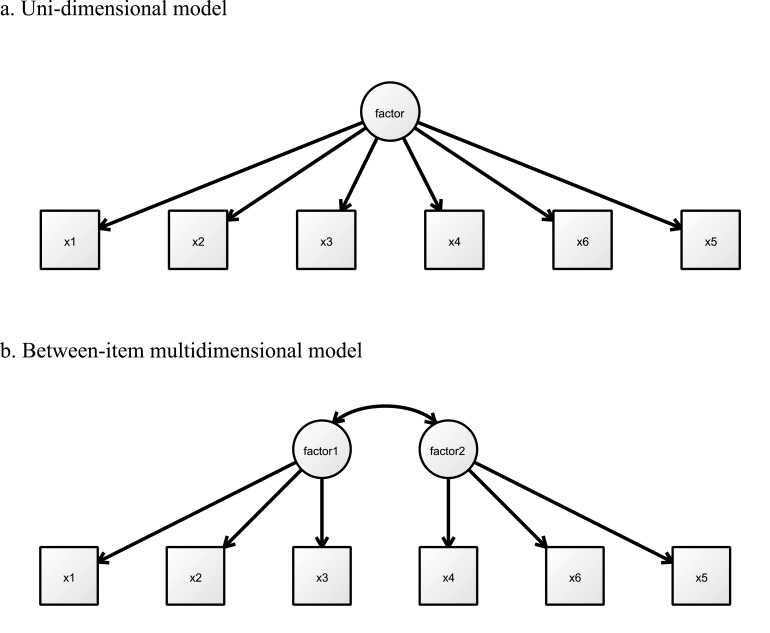
Two types of IRT models.

The statistical models used in our analyses are based on the multidimensional extension of a graded item response theory. We used multidimensional item response theory (MIRT) with maximum likelihood robust (MLR) in Mplus 7.2. Herein, we used the Monte Carlo integration algorithm. The MIRT estimated the item slope parameters and item difficulty parameters in the ABCR final item pool. In addition, the MIRT estimated the ABCR-14 total information curve (TIC), item information curve (IIC), and the item response category characteristic curve (IRCCC) that indicates the belief-conflict level at which a response in a given category or higher becomes probable. The MIRT was employed to estimate Akaike’s information criterion (AIC) and Bayesian information criterion (BIC). Then we compared two different IRT models with the 14 items for further consideration of the IRT model by AIC and BIC: (1) uni-dimensional model and (2) between-item multidimensional model (see Fig 1). Uni-dimensional models require a single factor dimension that is the most restrictive [41]. When an item can discriminate more factors, the assessment is said to be a between-item multidimensional model [42], which is the most general MIRT [43]. We assumed that ABCR is a between-item multidimensional model.

## Results

### Sample Characteristics

In this study total, 1683 nurses (57.1% response rate) participated, including 93.6% female, 6% male, and 0.4% other. Participants had licenses including 80.5% nursing, 3.3% midwife nurse, 9.2% public health nurse, 6.2% vocational nurse, and 0.9% others. Details on the study sample and JCQ score are reported in Table 1.

**Table 1:** Participant Characteristics (n = 1683).

Characteristics		Mean (SD)	%
**Gender**	Female		93.6
	Male		6.0
	Other		0.4
**Age**		35.9 (10.4)	
**Clinical experience**		13.0 (10.4)	
**License**	Nursing		80.5
	Midwife nurse		3.3
	Public health nurse		9.2
	Licensed Practical Nurse		6.2
	Other		0.9
**Position**	Head Nurse		4.6
	Assistant Head Nurse		4.1
	Chief nurse		6.5
	Staff		83.7
	Other		1.0
**Working arrangements**			
	Three shifts		34.2
	Two shifts		40.9
	Shift not involving three and two shifts		0.5
	Day shift		16.6
	Evening shift		4.1
	On call		1.7
	Other		1.9
**Vacation**	Two-day weekend		33.7
	Semimonthly two-day weekend		4.4
	Any two-day weekend		52.5
	Monthly two-day weekend		0.4
	Three times a monthly two-day weekend		1.8
	One-day weekend		0.1
	Other		7.1
**Taking a leave of absence**			
	Very good		9.6
	Good		58.1
	Fair		16.0
	Poor		9.9
	Neither agree nor disagree		6.3
**Health condition**			
	Very good		9.6
	Good		56.6
	Fair		22.5
	Poor		2.6
	Neither good nor bad		8.7
**Team Approach to Health Care**			
	Nutrition support team		9.8
	Respiratory care team		5.1
	Rehabilitation team		10.9
	Feeding and swallowing support team		4.8
	Diabetic Support team		5.9
	Decubitus care team		12.5
	Infection control team		12.6
	Emergency medicine team		5.7
	Palliative care team		9.3
	Medical safety team		11.7
	Other		11.7
**Marriage**	Yes		43.1
	No		56.9
**Smoking**	Yes		12.5
	No		87.5
**Drinking**	Yes		47.6
	No		52.4
**JCQ component scores**			
	Job demand	36.12 (5.247)	
	Skill discretion	35.69 (4.084)	
	Decision authority	33.42 (6.134)	
	Job control	69.11 (8.276)	
	Superior support	11.40 (2.705)	
	Coworker support	11.76 (2.134)	
	Social support	23.16 (4.272)	
	Job strain	0.5301 (0.1012)	

Note. SD = Standard Deviation

### Item reduction

As a result of the 45-item analysis, floor and ceiling responses were not observed in the distribution of answers (Table 2). Entropy value ranged from 2.007 to 2.635. Polyserial correlation coefficient between each item’s score and the total score ranged from 0.203 to 0.702. In these analyses, all items were not reduced.

**Table 2:** ABCR Item Analysis.

45 items of ABCR		Mean	SD	Entropy	PCC
Item 1	No matter how hard I try, I receive complaints from patients and their families.	3.088	1.501	2.483	0.509
Item 2	I am offended by the bad attitude of other staff.	3.693	1.646	2.635	0.624
Item 3	I experience difficulty because of the peculiar human interactions in my department.	3.829	1.589	2.621	0.641
Item 4	Other healthcare workers do not cooperate.	3.205	1.266	2.348	0.651
Item 5	I experience difficulty in caring for a patient with many demanding requests.	4.145	1.383	2.439	0.558
Item 6	I feel that I have a disagreement about response to patient.	3.763	1.198	2.290	0.609
Item 7	I feel that I fail to establish a trustworthy relationship with the patients and their families.	3.297	1.112	2.189	0.565
Item 8	I can empathize with a patient’s feelings and emotions, but I experience difficulty with unfair criticism.	4.154	1.433	2.455	0.603
Item 9	When I am very busy, I become impatient with the lengthy palaver of patients and their families.	4.144	1.369	2.424	0.539
Item 10	In my department, I do not experience mutual respect for others’ positions.	3.509	1.336	2.434	0.635
Item 11	Despite being a worker in the same department, I experience difficulty in understanding coworkers.	4.046	1.469	2.540	0.599
Item 12	I experience difficulties in the ineffectiveness of care.	4.152	1.224	2.271	0.477
Item 13	In general, the team members of this hospital understand the practice of other staff.	3.761	1.055	2.087	0.203
Item 14	I experience contradictory beliefs when I receive negative feedback from healthcare workers in the same department.	3.811	1.243	2.345	0.595
Item 15	I am unable to provide efficient services due to the lack of cooperation from other staff.	3.549	1.184	2.274	0.687
Item 16	Our workplace has good teamwork between healthcare workers and other staff.	3.794	1.047	2.090	0.361
Item 17	I experience stress due to strong, repeated negative complaints from patients and their families.	4.086	1.277	2.352	0.556
Item 18	I feel that I fail to establish a trustworthy relationship with other staff.	3.215	1.106	2.161	0.621
Item 19	When a team member is very busy, we support one another in the department.	2.964	1.099	2.094	0.312
Item 20	I experience a feeling of helplessness while caring for an afflicted patient.	4.042	1.225	2.294	0.423
Item 21	In the same department, we can perform well as a team.	3.659	1.048	2.067	0.450
Item 22	I have a difference of opinion with a superior authority.	3.677	1.282	2.362	0.576
Item 23	I am unable to suggest a valid opinion for an authoritarian workplace.	3.762	1.354	2.450	0.584
Item 24	I feel that I have not understood therapeutic strategies.	3.202	1.020	2.007	0.669
Item 25	I am tired of emotional suppression in front of patients and their families.	3.884	1.364	2.448	0.671
Item 26	I care for patients and their families who have doubts regarding healthcare service.	4.176	1.362	2.295	0.483
Item 27	Other staff members do not listen to my opinions.	3.326	1.274	2.364	0.650
Item 28	In this hospital, there is disagreement among healthcare workers.	2.975	1.276	2.330	0.666
Item 29	I feel I am misunderstood by the department supervisor.	3.355	1.403	2.472	0.691
Item 30	I hide my true emotions while caring for patients and their families.	4.177	1.298	2.347	0.545
Item 31	I feel lack of coherence as a team in my department.	3.304	1.241	2.315	0.667
Item 32	I experience difficulty in interpreting the frequent calls and complaints of the patients and their families.	4.382	1.550	2.594	0.443
Item 33	When the workplace is very busy, we can work as a team comprised of various staff.	3.888	1.164	2.220	0.282
Item 34	The other staff make unreasonable demands of me in the work.	3.606	1.316	2.408	0.575
Item 35	Despite busy days, I attentively listen to patients’ narratives.	4.449	1.262	2.203	0.309
Item 36	I experience difficulty in exerting myself in order to please the patient.	4.308	1.220	2.294	0.531
Item 37	I experience the lack of a healthy relationship at work.	3.352	1.191	2.232	0.613
Item 38	I cannot collaborate very well with staff whose thinking differs from mine.	3.493	1.201	2.270	0.701
Item 39	I am tired by forcibly pretending to be cheerful in front of the patient.	4.301	1.347	2.413	0.602
Item 40	I experience difficulty in developing collaborative relationships with different generations.	3.555	1.283	2.380	0.644
Item 41	I experience a communication problem between the supervisor and coworker in the same department.	3.640	1.338	2.435	0.660
Item 42	I have a hard time with teamwork due to non-cooperation from other staff.	3.262	1.163	2.229	0.702
Item 43	I experience difficulty in work while there is dissensus with other staff.	3.505	1.334	2.422	0.663
Item 44	I feel that other staff members dodge responsibility by shifting blame to me.	2.997	1.455	2.474	0.658
Item 45	The healthcare workers of the hospital have poor mutual relationships.	2.890	1.264	2.277	0.702

Note. SD = Standard Deviation, PCC = Polyserial Correlation Coefficient

### Construct validity

EFA determined the underlying factor structure of the item set. The three factors and 14 items were retained after the EFA procedure, as displayed in Table 3. ABCR-14 provided an optimal fit to the data (RMSEA = 0.067 [0.061–0.073]; CFI = 0.986; TLI = 0.976).

**Table 3:** Construct Validity of ABCR-14.

Items of ABCR-14	Factor 1	Factor 2	Factor 3	UF
Item 40	**0.800** *	-0.011	0.070*	0.334
Item 42	**0.795** *	0.135*	-0.003	0.237
Item 37	**0.776** *	-0.021	0.006	0.413
Item 41	**0.770** *	0.012	0.037*	0.380
Item 31	**0.745** *	0.088*	-0.017	0.375
Item 43	0.110*	**0.795** *	-0.035	0.285
Item 38	0.137*	**0.738** *	-0.001	0.331
Item 34	-0.002	**0.706** *	0.021	0.492
Item 27	0.063	**0.684** *	0.038*	0.461
Item 2	-0.066	**0.669** *	0.142*	0.513
Item 8	-0.039	0.077*	**0.779** *	0.364
Item 17	0.033	-0.031	**0.788** *	0.381
Item 9	0.007	0.055	**0.719** *	0.449
Item 5	0.064*	-0.009	**0.740** *	0.427
**Geomin factor correlation**				
Factor 1	1.000			
Factor 2	0.532*	1.000		
Factor 3	0.279*	0.352*	1.000	
**Model fit information**				
RMSEA	0.067 [90%CI = 0.061–0.073]			
CFI	0.986			
TLI	0.976			

Note. UF = Unique Factor

* = Significant at 5% level

CI = Confidence Interval, Factor 1 = belief conflict among the same healthcare workers; Factor 2 = belief conflict between healthcare workers and other staff; Factor 3 = belief conflict in therapeutic relationships

### Structural validity

Based on the EFA, an analysis of ABCR-14 was tested with CFA. The result showed that this model provided an optimal fit to the data (Table 4) (RMSEA = 0.061 [0.056–0.066]; CFI = 0.984; TLI = 0.980).

**Table 4:** Structural Validity of ABCR-14.

	Estimate	S.E.	Est./S.E.	Two-TailedP-Value	90% CI
**Latent variables**					
Factor 1					
Item 40	0.807	0.009	91.672	0.000	0.793; 0.822
Item 42	0.889	0.007	120.204	0.000	0.876; 0.901
Item 37	0.746	0.011	67.722	0.000	0.728; 0.764
Item 41	0.781	0.01	81.338	0.000	0.765; 0.797
Item 31	0.792	0.01	77.846	0.000	0.775; 0.809
Factor 2					
Item 43	0.844	0.009	91.117	0.000	0.828; 0.859
Item 38	0.834	0.009	92.14	0.000	0.819; 0.849
Item 34	0.691	0.013	53.569	0.000	0.669; 0.712
Item 27	0.736	0.012	61.808	0.000	0.716; 0.756
Item 2	0.669	0.015	45.561	0.000	0.644; 0.693
Factor 3					
Item 8	0.794	0.012	68.106	0.000	0.775; 0.813
Item 17	0.769	0.012	61.688	0.000	0.749; 0.790
Item 9	0.753	0.013	57.095	0.000	0.731; 0.775
Item 5	0.764	0.013	58.697	0.000	0.742; 0.785
**Factor correlation**					
Factor 2					
Factor 1	0.639	0.014	44.531	0.000	0.615; 0.662
Factor 3					
Factor 1	0.345	0.022	15.765	0.000	0.309; 0.381
Factor 2	0.426	0.022	19.809	0.000	0.391; 0.462
**Model fit information**						
RMSEA	0.061 [90% CI = 0.056–0.066]					
CFI	0.984				
TLI	0.980				

Note. CI = Confidence Interval, Factor 1 = belief conflict among the same healthcare workers; Factor 2 = belief conflict between healthcare workers and other staff; Factor 3 = belief conflict in therapeutic relationships

### Hypothesis testing

ABCR-14 had a high level of convergent and discriminant validity, as shown in Table 5. Excellent convergent and discriminant validity were demonstrated in relation to evaluations.

**Table 5:** Hypothesis Testing of ABCR-14.

ABCR-14	AVE	SCC		
		Factor1	Factor2	Factor3
Factor 1	0.647	1.000		
Factor 2	0.574	0.408	1.000	
Factor 3	0.593	0.119	0.181	1.000

Note. AVE = Average Variance Extracted; SCC = squared correlation coefficient; Factor 1 = belief conflict among the same healthcare workers; Factor 2 = belief conflict between healthcare workers and other staff; Factor 3 = belief conflict in therapeutic relationships

### Internal consistency reliability

Internal consistency reliability coefficients of ABCR-14 (total score and all subscales) were all in the acceptable range, from 0.831 and 0.884 (Table 6).

**Table 6:** Internal Consistency Reliability of ABCR-14.

	Mean	SD	Cronbach’s Alpha
ABCR-14			
Total	51.26	11.641	0.882
Factor 1	17.11	5.147	0.884
Factor 2	17.62	5.357	0.846
Factor 3	16.53	4.457	0.831

Note. SD = Standard Deviation, Factor 1 = belief conflict among the same healthcare workers; Factor 2 = belief conflict between healthcare workers and other staff, Factor 3 = belief conflict in therapeutic relationships

### Concurrent validity

ABCR-14 was confirmed by the correlation between ABCR-14 factor scores and the JCQ. The same professional belief conflict of ABCR-14 factor scores shows a strong negative correlation among JCQ *supervisor support*, *coworker support*, and *workplace social support*. The ABCR-14 factor scores also show a weak positive association between *job strain* and *job demand* on the JCQ. Details on concurrent validity are reported in Table 7.

**Table 7:** Concurrent Validity of ABCR-14 and JCQ.

	JCQ							
ABCR-14	JD	SD	DA	JC	SuS	CS	SoS	JS
Factor 1	0.143**	-0.109**	-0.188**	-0.144**	-0.420**	-0.516**	-0.525**	0.238**
Factor 2	0.195**	-0.033	-0.135**	-0.069**	-0.291**	-0.348**	-0.348**	0.222**
Factor 3	0.203**	-0.020	-0.087**	-0.019	-0.146**	-0.156**	-0.171**	0.182**

Note. PC = Participant characteristics; JD = Job demand; SD = Skill discretion; DA = Decision authority; JC = Job control; SuS = Superior support; CS = Coworker support; SoS = Social support; JS = Job strain

* = Significant at 5% level;

** = Significant at 1% level;

Factor 1 = belief conflict among the same healthcare workers; Factor 2 = belief conflict between healthcare workers and other staff; Factor 3 = belief conflict in therapeutic relationships

### Item response

All ABCR-14 items’ parameters were estimated using the MIRT. The information criteria clearly supported the between-item multidimensional model (AIC = 66379.782, BIC = 66857.475) over the uni-dimensional model (AIC = 70015.450, BIC = 70476.859), so that the between-item multidimensional model was more appropriate for describing the ABCR-14 than the uni-dimensional model.

As Table 8 shows, items were indicated based on the magnitude of the slope parameter, item difficulty parameters, and information criteria. S1 Fig presents the TIC plots for all three factors. The results showed ABCR-14 to be broadly applicable from mild to severe belief conflict; factor 1 provided much more information than the other factors. S2 Fig presents the IIC plots for all 14 items of each factor. Items 8, 42, and 43 provided more information than other items. The result of IRCCC appears in S3 to S5 Figs. The ABCR-14 design was based on a 7-point Likert scale. As S2 Fig indicates, response categories 3, 4, and 5 were more likely to be selected around the mean of the trait (θ = 0). However, some item response categories were redundant with points 4 or 6.

**Table 8:** Item Response on ABCR-14.

Items of ABCR-14	α	β1	β2	β3	β4	β5	β6
**Factor 1**							
Item 40	1.489	-1.598	-0.717	0.057	0.753	1.535	2.04
Item 42	1.722	-1.445	-0.537	0.227	1.147	1.886	2.409
Item 37	1.402	-1.662	-0.608	0.087	1.127	1.777	2.171
Item 41	1.435	-1.638	-0.713	0.012	0.663	1.435	1.941
Item 31	1.527	-1.572	-0.537	0.198	1.041	1.73	2.17
**Factor 2**							
Item 5	1.253	-1.78	-1.006	-0.422	0.088	1.102	1.894
Item 8	1.419	-1.65	-0.911	-0.403	0.041	1.087	1.793
Item 9	1.270	-1.766	-1.052	-0.401	0.09	1.121	1.865
Item 17	1.325	-1.721	-1.076	-0.455	0.21	1.262	2.087
Item 2	1.424	-1.646	-0.635	-0.143	0.256	1.081	1.573
**Factor 3**							
Item 27	1.473	-1.61	-0.62	0.062	0.842	1.689	2.263
Item 34	1.236	-1.794	-0.764	-0.093	0.573	1.495	2.055
Item 38	1.384	-1.676	-0.727	-0.058	0.821	1.69	2.125
Item 43	1.558	-1.551	-0.636	-0.015	0.682	1.466	1.964
**Information criteria**							
**AIC**	66379.782						
**BIC**	66857.475						

Note. α = Item slope parameters; β = Difficulty parameters; AIC = Akaike’s Information Criterion; BIC = Bayesian information criterion; Factor 1 = belief conflict among the same healthcare workers; Factor 2 = belief conflict between healthcare workers and other staff; Factor 3 = belief conflict in therapeutic relationships

## Discussion

### Psychometric properties of the ABCR-14

We developed and examined the psychometric properties of ABCR-14 as a new, belief-conflict-specific, self-reported measure for evaluating nurses. Overall, the validity and reliability of ABCR-14 were satisfactory. To the best of our knowledge, this is the first study on the development and psychometric properties of an assessment scale for belief conflict.

Several results suggested evidence for the validity and reliability of ABCR-14 (see Tables 3 to 7). The construct and structural validity assessed by EFA and CFA were acceptable for the 14 items consisting of a three-factor structure (Tables 3 and 4). That is, ABCR-14 has goodness of fit indices to the validity of the factor. Table 5 shows that hypothesis testing of this study’s result was acceptable for ABCR-14’s convergent and discriminant validity. These results are identical to that of the DAB-based framework concept of ABCR. This fact clearly proves that the model fits the data. Assessed by Cronbach’s alpha, internal consistency reliability was acceptable for the 14 items consisting of the three-factor structure (Table 6). ABCR-14 was found to show good reliability and therefore has high validity and reliability.

Additionally, concurrent validity was assessed by comparison of ABCR-14 and JCQ participant characteristics (including *leave of absence* and *health condition*) (Table 7). A modest correlation (0.182 from 0.238) between ABCR-14 and JCQ *job strain* was indicated. Job strain refers to a combination of job demand and job control. Job demand refers to the amount, pace, and difficulty of the work, while job control refers to the ability to make work-related decisions or be creative at work. ABCR-14 has a modest connection with *work-related stressor* and a deep negative connection with JCQ *support*. Specifically, the same professional belief conflict of the factor in ABCR-14 has strongly negative connection with JCQ *support*, which refers to assistance received from co-workers and supervisors. This result shows that ABCR-14 is associated with interpersonal problems and poor teamwork in a workplace. Thus, we can say with fair certainty that this result shows an association between belief conflict and job stress.

The MIRT was used to examine individual item characteristics. From the results of information criteria, we concluded that the hypothesis of a between-item multidimensional model was supported. The result of the MIRT resembled that of results of various validities. The ABCR-14 scores proved to have high item slope parameters, in the range of 1.236 and 1.722. Difficulty parameter scores were wide, ranging from -1.794 to 2.409. These data offered strong support for good item response of item slope parameters and difficulty parameters (Table 8). The ABCR-14 amount of information was sufficiently identified (see S1 and S2 Figs). However, the evidence for supporting this model’s information criterion and IRCCC might be exiguous (see S3 to S5 Figs). ABCR-14 item design was based on a 7-point Likert scale. This scale might need to be explored from the MIRT point of view. Yet, as mentioned above, there is enough evidence to show that ABCR-14 has high validity and reliability. From this viewpoint, one may state that the ABCR-14 Likert scale design is correct.

### Practice implications

There are some practical implications for developing a scale to measure nurses’ belief conflict to clarify the problems arising from belief conflict. Valid and reliable assessment is needed for designing and evaluating management programs based on DAB. Interventions to resolve belief conflicts can target the beliefs that guide conflict behavior among nurses and other healthcare practitioners. In other words, ABCR-14 could lead to development of programs effective in achieving collaboration among team members. Additionally, this assessment provides individual belief conflict assessment and clarification of problems and improvements in multidisciplinary care. Particularly, healthcare providers who work with job management can use ABCR-14 to identify problems that prevent positive collaboration and, in turn, intervene to delay or prevent belief conflict.

For example, if nurses have high risk of belief conflict with patients’ families from the result of ABCR-14, we should perform an analysis on the belief conflict’s structure. Analysis of belief conflict by DAB involves the following questions: why, who, when, how, and where? What are the causes of the belief conflict? We have used data from unstructured assessment to analysis of nurse with belief conflict: including narrative interviewing and participant observations with the nurse and patients’ families. After analyzing belief conflict through ABCR-14, we should choose and practice the best dissolution of the problem by following these DAB rules: 1) Accomplishing a goal is the first priority, 2). The method is a means to an end. 3) The effectiveness of a method depends on the circumstance and goal, and the method’s effectiveness is judged ex post facto. To practice these rules, we reflect deeply on a circumstance and purpose of nurse and patients’ families, verify the team’s condition in practice, and reconfigure the team’s common goal. These can be held at a meeting to discuss common circumstance and purpose. Or, we can talk about common circumstance and purpose in a natural situation. In addition, we can optimize the method of achieving the goal and become able to work constructively together. The method of achieving a purpose is able to give by chose the most practical option: including stepwise and realistic goal setting, purpose-designed planning, increases options, unbiased consideration, unconventional thinking, meditation, reflection on the action, and evidence-based practice. Thus, the result of ABCR-14 can shape the direction of DAB-based practice.

### Limitations

This study had a few limitations. For example, to reduce participant burden, we did not perform test-retest reliability. Next the survey was conducted at only 11 hospitals, raising the issue of the findings’ generalizability. Furthermore, the study did not investigate healthcare practitioners other than nurses. However, based on the findings of this study, ABCR-14 is a potentially useful tool for estimating belief-conflict-related problems and for monitoring stress-related problems as part of team management.

## Conclusions

Overall, the study findings suggested that ABCR-14 is a valid, reliable scale for assessing nurses’ belief conflicts. Further studies are recommended to confirm its application in clinical practice.

## Supporting Information

S1 Fig TIC of ABCR-14. (DOCX)

S2 Fig IIC of ABCR-14. (DOCX)

S3 Fig IRCCC of Factor 1 in ABCR-14. (DOCX)

S4 Fig IRCCC of Factor 2 in ABCR-14. (DOCX)

S5 Fig IRCCC of Factor 3 in ABCR-14. (DOCX)
