# Supplementary material for: Development of the Assessment of Belief Conflict in Relationship-14 (ABCR-14)
Source: PLoS One. 2015 Aug 6;10(8):e0129349. doi: 10.1371/journal.pone.0129349 (PMC4527743; doi:10.1371/journal.pone.0129349)
Supplement: S3 Fig — (DOCX) [file pone.0129349.s003.docx]

**Appendix**

**S3 Figure. IRCCC of Factor 1 in ABCR-14**

Note. Factor 1 = belief conflict among the same healthcare workers
